# Novel BTK Mutation in Patient with Late Diagnosis of X-Linked Agammaglobulinemia

**DOI:** 10.1155/2023/6698913

**Published:** 2023-11-25

**Authors:** Amanpreet Kalkat, Olivia Humpel, Robert Hostoffer

**Affiliations:** ^1^University Hospitals of Cleveland, 5915 Landerbrook Dr, Mayfield Heights, OH 44124, USA; ^2^Lake Erie College of Osteopathic Medicine, 5000 Lakewood Ranch Boulevard, Bradenton, FL 34211, USA

## Abstract

X-linked agammaglobulinemia (XLA) is a genetic disorder with mutation in Bruton's tyrosine kinase (BTK). Defects in B cell development and immunoglobulin production lead to recurrent infections following loss of maternal IgG at 6 months of age. A 55-year-old male with a longstanding common variable immunodeficiency diagnosis on infusion therapy presented to the clinic with cutaneous T-cell lymphoma, which inspired overall repeat evaluation. Immunoglobulin levels and lymphocyte markers, family history, and genetic testing prompted a true diagnosis of XLA and novel mutation in the BTK gene. Disease-associated mutations have been noted in all five domains of BTK, with missense variants most commonly cited among the 100s of reported genetic alterations. The BTK protein is expressed in hematopoietic lineages and plasma cells, with the exception of T lymphocytes. Disruption in the protein function or absence of BTK halts normal B cell development at the pre-B transitional cell stage and induces premature apoptosis. We present the first reported case of a novel hemizygous BTK c.1492C > G mutation in a patient causing XLA.

## 1. Introduction

X-linked agammaglobulinemia (XLA) is a genetic disorder with mutation in Bruton's tyrosine kinase (BTK) with X-linked recessive inheritance that leads to defects in B cell development. Patients with XLA have decreased numbers of mature B cells in peripheral blood and a lack of all immunoglobulin isotypes. This prevents patients from creating immunoglobulins and allows for the preponderance of recurrent upper and lower respiratory tract infections after 6 months of age when the maternal transplacental IgG is consumed [1]. We present the first reported case of a novel hemizygous BTK c.1492C > G mutation in a patient causing XLA.

## 2. Case Presentation

**Figure 1:**
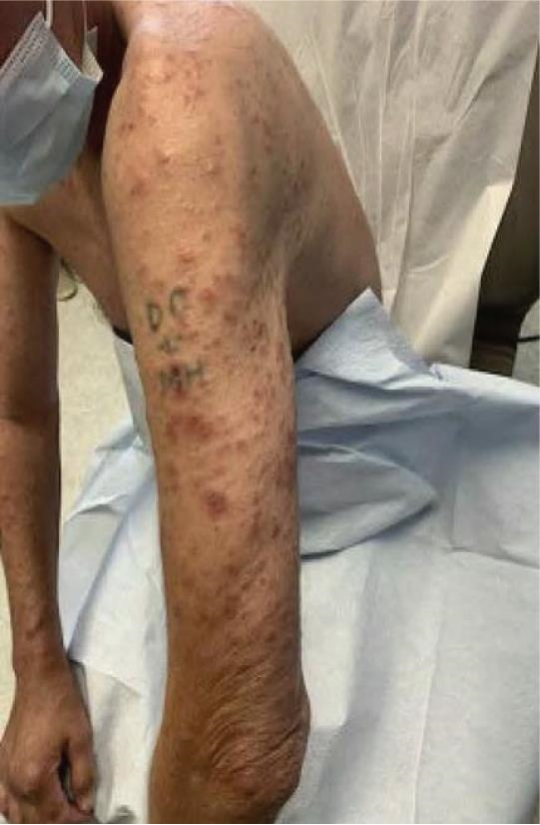
Clinical dermatological appearance of edematous erythematous papules on the arms.

**Figure 2:**
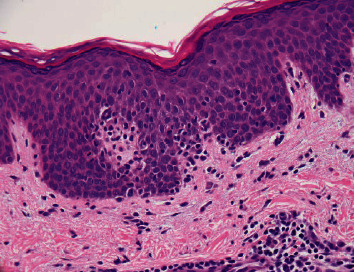
Exocytosis of small lymphocytes in the epidermis with tagging of the basement membrane zone.

**Figure 3:**
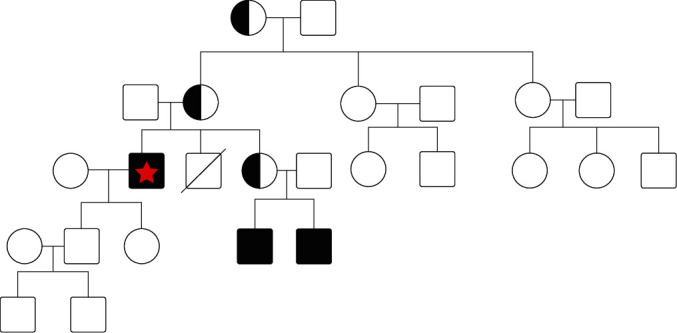
Pedigree showing X-linked recessive inheritance pattern. Red star indicates the patient. Given that only his mother and grandmother are carriers of a mutation for XLA, the only affected individuals are male (the patient and his nephews).

A 55-year-old male with a longstanding misdiagnosis of common variable immunodeficiency presented with edematous erythematous papules on the nose and arms with swollen lymph nodes (Figure 1). Biopsy revealed exocytosis of small lymphocytes in the epidermis with tagging of the basement membrane zone (Figure 2). FDG-PET scan confirmed a diagnosis of cutaneous T-cell lymphoma, mycosis fungoides type. Immunoglobulin levels and lymphocytes markers were drawn at this time (Table 1). The patient's history of recurrent infections since childhood and lack of CD19+ B cells and serum immunoglobulins align with a classical diagnosis of XLA. Genetic testing revealed a novel hemizygous Leu498Val (c.1492C > G) mutation in the BTK gene leading to a correct diagnosis of XLA. The identification of the novel mutation and new diagnosis prompted further investigation into the patient's family history (Figure 3). The patient's nephews require immunoglobulin replacement therapy for recurrent infections, and genetic testing was recommended although not performed.

## 3. Discussion

The gene encoding BTK has been mapped to the long arm of the X chromosome Xq21.3–q22 and consists of 19 exons. BTK belongs to the Tec family of nonreceptor tyrosine kinases [2]. The BTK protein is 659 residues long and is expressed in hematopoietic lineages, except for T lymphocytes and plasma cells. Mutations in all five domains of BTK have been noted to cause disease (PH, TH, SH1, SH2, and SH). CpG sites forming arginine residues are most frequently affected [3]. The absence of BTK halts normal B cell development at the pre-B transitional cell stage with premature induction of apoptosis. Over 600 unique molecular events have been cited. Missense mutations are the most common (40%), followed by deletions (20%), nonsense (17%), split site mutations (16%), and insertions (7%) [4]. Genetic testing for this patient revealed a novel mutation that replaced leucine with valine. This missense variant was expected to disrupt BTK protein function (invitae).

Diagnosis of XLA in atypical or sporadic cases may be delayed. In 30%–50% of symptomatic patients, there is no obvious family history of the disease [5]. Several reports have described misdiagnosis with CVID and later discovery of BTK mutations prompting reclassification. One study reported five patients previously diagnosed with CVID who were later reclassified as atypical XLA variants [5]. Variation in the effects of different BTK mutations on function protein production may explain small numbers of immunoglobulin producing CD19+ B cells. A marginal ability to evade severe infection through childhood may contribute to late diagnosis [5].

XLA patients are treated with monthly infusions or weekly subcutaneous infusions of gamma globulin. While infusion therapy decreases risk of infection, different reports quote a 1.5%–2% risk of malignancy among patients with XLA [6]. The mechanism of carcinogenesis in XLA is unclear and the role for chronic infections and chronic inflammation has been suggested [7].

## 4. Conclusion

The patient's childhood history of recurrent infections and reported family history indicate an X-linked recessive inheritance pattern. Initial diagnosis with CVID was due to the patient's history and agammaglobulinemia. Occurrence of cutaneous T-cell lymphoma inspired repeat general evaluation, which implicated a diagnosis of XLA given an absence of CD19+ B cells, agammaglobulinemia, and a mutation in the BTK gene. We report a novel hemizygous BTK Leu498Val (c.1492C > G) mutation in an XLA patient formerly misdiagnosed with CVID due to the phenotypic agammaglobulinemia.

## Figures and Tables

**Table 1:** Immunoglobulin levels were drawn and revealed baseline agammaglobulinemia while on immunoglobulin replacement therapy, as well as a lack of serum CD19+ B cells.

	Immunoglobulin levels	Reference range
IgA	7	70–400 mg/dL
IgM	<10	40–230 mg/dL
IgG	644	700–1,600 mg/dL

	Lymphocyte markers	Reference range

CD3	0.581	0.71–4.18
CD4	0.154	0.35–2.75
CD4/CD8	0.38	1–3.5
CD19	Absent	—

## Data Availability

Data are available on National Center for Biotechnology Information. ClinVar; [VCV000530950.7], (https://www.ncbi.nlm.nih.gov/clinvar/variation/VCV000530950.7).
